# Defining the conformational landscape of glycogen synthase kinase 3 beta (GSK3β)

**DOI:** 10.1107/S2059798326010533

**Published:** 2026-10-23

**Authors:** Akanksha Gurtu, Fernanda Laezza

**Affiliations:** a Department of Pharmacology and Toxicology, The University of Texas Medical Branch, Galveston, TX77555, USA; b Sealy Center for Environmental Health and Medicine, The University of Texas Medical Branch, Galveston, TX77555, USA; University of Queensland, Australia

**Keywords:** glycogen synthase kinase 3 beta, GSK3β, X-ray crystallography, kinase conformations, DFG-in

## Abstract

Glycogen synthase kinase 3 beta (GSK3β) is a serine/threonine kinase implicated in Alzheimer’s disease, type 2 diabetes, oncology and bipolar disorder, and represents an extensively pursued therapeutic target. Despite the substantial accumulation of GSK3β crystal structures in the Protein Data Bank (PDB), no systematic consolidation and conformational analysis of this dataset have been performed. Here, we manually classified all deposited GSK3β crystal structures, comprising 111 unique PDB entries and 88 distinct co-crystallized ligands (data extracted March 11th, 2026). Using the pre-existing KinCoRe dataset and standardized nomenclature, we assigned conformational states across the complete GSK3β structural ensemble and evaluated their relationships with bound ligands. All structures adopt the DFG-in kinase conformation but populate eight distinct conformational substates defined by combinations of activation-loop geometry, αC-helix orientation, regulatory salt-bridge status, αC–β5-loop and phosphorylation state. Our structural analysis reveals that chemically distinct Type I inhibitors stabilize distinct conformational substates, including variation in activation-loop arrangement and activity-like states. These relationships have not previously been characterized across the complete GSK3β crystal structure ensemble. This work provides the first comprehensive conformational landscape of resolved GSK3β crystal structures, a curated structural reference to guide the selection of potentially biologically relevant conformations for structure-based drug design and a systematic framework for analogous kinase-wide conformational analyses.

## Abstract 2

This article presents a systematic structural curation of all deposited GSK3β crystal structures to map its conformational ensemble, revealing eight distinct conformational states.

## 1. Introduction

### 1.1. Kinase structural conformations as a basis for selective drug discovery

Protein kinases participate in the regulation of virtually every major cellular signaling pathway and are among the most intensively pursued therapeutic target classes (Attwood *et al.*, 2021 ▸; Hanks & Hunter, 1995 ▸). To date, more than 90 kinase inhibitors have received FDA approval (Roskoski, 2026 ▸). However, achieving selectivity across the ∼538-member human kinome remains a central challenge, as most approved inhibitors engage the highly conserved ATP-binding site (Duong-Ly & Peterson, 2013 ▸; Gavrin & Saiah, 2013 ▸; Manning *et al.*, 2002 ▸; Roskoski, 2026 ▸). Off-target kinase inhibition can limit therapeutic utility and contribute to adverse effects (Bayazeid & Rahman, 2021 ▸).

Kinase conformational plasticity provides opportunities to improve selectivity. Conformationally selective inhibitor design offers one route to overcome the limitations imposed by kinome-wide ATP-site conservation (Anderson *et al.*, 2024 ▸; Byrne *et al.*, 2020 ▸; Hari *et al.*, 2014 ▸; Westwood *et al.*, 2009 ▸). By preferentially recognizing a conformational state, cryptic pocket or allosteric site that is less conserved or differentially accessible across kinases, an inhibitor may discriminate between the intended kinase target and off-target kinases. Traditionally, small-molecule kinase inhibitors are classified by their binding mode. Type I inhibitors occupy the ATP-binding site, typically in DFG-in conformations, whereas Type I^1/2^ inhibitors extend from the ATP pocket into an adjacent region. Type II inhibitors recognize DFG-out conformations and occupy both the ATP site and the adjacent hydrophobic back pocket. Type III inhibitors bind allosteric sites outside the ATP-binding cleft. Kinase inhibitors, such as Type II or Type III, that stabilize the DFG-out conformation engage less conserved pockets than the ATP-binding site and therefore have the potential to achieve improved kinome selectivity (Fang *et al.*, 2013 ▸; Vijayan *et al.*, 2015 ▸).

Additionally, knowledge of the conformational landscape of a kinase is essential for reliable structure-based virtual screening and docking. A compound may bind only when the kinase adopts a particular activation-loop arrangement, αC-helix position or DFG configuration, and a single crystal structure may therefore fail to represent a ligand-compatible conformation. Conversely, an experimentally curated ensemble of kinase conformations enables multiple conformation-based virtual screening (ensemble docking) and supports the selection of target structures that are appropriate for the intended binding mode (Chen *et al.*, 2026 ▸; Da *et al.*, 2023 ▸; Park *et al.*, 2009 ▸; Ricci-Lopez *et al.*, 2021 ▸; Tian *et al.*, 2014 ▸).

A prerequisite for rational structure-guided design of selective inhibitors or multiple conformation-based virtual screening is a complete understanding of the conformational landscape and a curated conformational ensemble. Here, we address this gap for glycogen synthase kinase 3 beta (GSK3β), a critical kinase in neurodegenerative disease, metabolic dysfunction and cancer (Baumgartner *et al.*, 2025 ▸; Daoud & Taha, 2026 ▸; Hamzé *et al.*, 2022 ▸; Mandlik *et al.*, 2024 ▸; Marosi *et al.*, 2022 ▸), by systematically cataloging all publicly available structural data based on conformational states.

### 1.2. GSK3β as a case study for conformational classifications

GSK3β is a serine/threonine protein kinase implicated in several biological pathways. The kinase was initially identified through its role in regulating glycogen synthase and insulin signaling (Welsh & Proud, 1993 ▸). GSK3β subsequently emerged as a key component of Wnt/β-catenin signaling, developmental cell-fate regulation, and metabolic and inflammatory pathways (Hoffmeister *et al.*, 2020 ▸; Hur & Zhou, 2010 ▸; Jing *et al.*, 2008 ▸; Kwon *et al.*, 2014 ▸; Liu *et al.*, 2002 ▸). Its dysregulation has been implicated in type 2 diabetes, cancer, neurogenerative disease and psychiatric disorders. GSK3β has also been linked to tau hyperphosphorylation in Alzheimer’s disease and has been long connected to the mechanism of action of lithium in bipolar disorder (Ishiguro *et al.*, 1992 ▸; Jope, 1999 ▸; Klein & Melton, 1996 ▸; Li & Jope, 2010 ▸; Mandelkow *et al.*, 1992 ▸; Takashima *et al.*, 1998 ▸).

Despite its therapeutic potential, GSK3β remains difficult to selectively modulate. GSK3β has more than 100 reported substrates, and broad or indiscriminate inhibition may therefore disrupt physiological functions that are distinct from those driving disease (Beurel *et al.*, 2015 ▸; Sutherland, 2011 ▸). In addition, the high similarity of the GSK3β catalytic domain to related kinases limits selectivity through traditional kinase inhibitor ATP-site recognition (Bhat *et al.*, 2018 ▸; Eldar-Finkelman & Ilouz, 2003 ▸; Huisamen *et al.*, 2016 ▸). Thus, many ATP-competitive GSK3β inhibitors face both kinome-wide off-target activity and limited selectivity. Additionally, drug resistance remains a challenge for GSK3β inhibitors (Arciniegas Ruiz & Eldar-Finkelman, 2021 ▸; Turkistani *et al.*, 2024 ▸; Wang *et al.*, 2025 ▸). These factors, together with pathway-dependent toxicity and the potential consequences of prolonged canonical pathway inhibition, have constrained clinical development despite extensive medicinal chemistry effort (Glibo *et al.*, 2021 ▸; Hall *et al.*, 2015 ▸; Huisamen *et al.*, 2016 ▸; Wang *et al.*, 2025 ▸).

These challenges establish a strong rationale for defining the conformational landscape of GSK3β. Conformationally selective inhibitors, allosteric inhibitors, substrate-competitive inhibitors, protein–protein interaction inhibitors and targeted protein-degradation strategies have all been proposed as approaches to improve the therapeutic precision of GSK3β modulation (Balasubramaniam *et al.*, 2020 ▸; Balboni *et al.*, 2023 ▸; Buch *et al.*, 2010 ▸; Holmqvist *et al.*, 2025 ▸; Singh *et al.*, 2026 ▸). The rational development and optimization of these strategies require knowledge of conformational states, regulatory features and ligand chemotypes associated with particular states. Although extensive prior work has characterized GSK3β inhibitor-binding regions and individual ligand-bound structures (Ganai *et al.*, 2025 ▸; Kramer *et al.*, 2012 ▸; Wagner *et al.*, 2016 ▸), the complete set of deposited GSK3β structures has not been systematically consolidated into a single conformational framework. Consequently, the structural diversity captured across the available crystal structure ensemble, the relationships between GSK3β conformations and bound ligands, and the conformational states absent from the current structural data remain undefined.

To address this gap, we manually classified all available GSK3β crystal structures using the KinCoRe dataset and standardized nomenclature. This framework distinguishes beyond broad active- or inactive-like states to identify finer conformational subclasses using the geometry of the DFG motif, the αC-helix orientation and other functionally relevant structural features. We use this curated structural dataset to address three questions. (i) What conformational states of GSK3β have been captured and what states are missing from the landscape? (ii) How do the existing GSK3β conformational states compare with one another? (iii) Are there unique sub-conformational states that researchers need to consider for drug discovery? By defining the experimentally observed conformational landscape of GSK3β, this study provides a structural reference for selecting conformations for docking experiments, guidance for structure-based drug design and a systematic framework for analogous analyses of other kinases.

The eight unique conformations identified from this study can support selection of GSK3β structures for docking based on ligand class and intended binding mode, facilitate ensemble virtual screening across structurally distinct target states, aid the interpretation of structure–activity relationships and help prioritize conformations for ligand optimization. Importantly, the classification does not itself establish that a given state is therapeutically selective; rather, it provides the experimentally grounded conformational ensemble required to formulate and test such hypotheses.

### 1.3. Structural features of GSK3β that define its conformation

GSK3β exists as a homodimer, forming homotypic head-to-tail dimers noted in crystallized structures (Dajani *et al.*, 2003 ▸; Lowe *et al.*, 1997 ▸; ter Haar *et al.*, 2001 ▸). Following typical kinase domains and structures, GSK3β contains a bilobal fold divided into the N-terminal lobe, containing seven-stranded β-sheets (β1–β7) and a conserved αC helix, and the C-terminal lobe (Fig. 1 ▸*a*). The N- and C-terminal lobes of GSK3β are connected through a hinge region (residues 134–137) whose backbone forms an anchoring region for ATP and kinase inhibitors. Each lobe contains key conserved, regulatory regions that mediate the primary kinase function of GSK3β (Figs. 1 ▸*b* and 1 ▸*c*).

The N-terminal lobe of GSK3β contains a conserved glycine-rich loop (G-loop, also referred to as the phosphate binding loop or P-loop), valine–alanine–isoleucine–lysine (VAIK) motif and αC helix (Fig. 1 ▸*b*). The flexible G-loop, situated between the β3 and β4 strands (residues 62–70), binds and positions the γ-phosphate of ATP to facilitate phosphoryl transfer. The glycine residues allow flexibility to clamp or release ATP during catalysis (Bossemeyer, 1994 ▸; Saraste *et al.*, 1990 ▸). The VAIK motif follows the G-loop (in the β5 strand, residues 82–85) and contains the catalytic lysine, Lys85, which forms a salt bridge with an acidic residue in the conserved αC helix (Fig. 1 ▸*b*). The αC helix of GSK3β (residues 95–104) includes Arg96, which coordinates substrate hydroxyl deprotonation for phosphoryl transfer (Frame *et al.*, 2001 ▸), and the key acidic residue Glu97, which stabilizes the kinase and is a determinant of catalytic activity (Fig. 1 ▸*b*). In the enzymatically active state, GSK3β forms an electrostatic bond between Lys85 and Glu97. The absence of this bond indicates an enzymatically inactive GSK3β (Buescher & Phiel, 2010 ▸).

The C-terminal lobe of GSK3β contains several helices (αD–αO, αEF) and two short β-sheets (β8–β9) (Fig. 1 ▸*a*). This lobe contains the activation segment comprised of a catalytic loop (C-loop) and activation loop. The C-loop (residues 182–186) is flanked by the conserved His–Arg–Asp (HRD) motif of GSK3β (residues 179–181), which functions as a catalytic base to deprotonate the substrate hydroxyl group and coordinate phosphate transfer (Fig. 1 ▸*b*). The activation loop of GSK3β (residues 200–226) is a critical substrate-binding region containing the Asp–Phe–Gly (DFG) and Ala–Pro–Glu (APE) motifs (Fig. 1 ▸*b*). The DFG motif binds magnesium ions (cofactor) and coordinates β- and γ-phosphates in the ATP-binding cleft (Pandey & DeGrado, 2016 ▸; Rajamani *et al.*, 2026 ▸). Phe201 in the DFG motif forms the hydrophobic spine (Met101, Leu112, His179 and Phe201) which stabilizes the active conformation of GSK3β (Buch *et al.*, 2010 ▸). The APE motif acts as a structural anchor to stabilize the C-terminal lobe and coordinate substrate interactions. Located within the activation loop is the Tyr216 residue, phosphorylation of which has been shown to increases the catalytic activity of GSK3β and allow substrate access to the substrate-binding pocket (Buescher & Phiel, 2010 ▸; Hughes *et al.*, 1993 ▸). Lastly, the C-terminal lobe contains the axin-binding domain (ABD; esidues 262–299; Fig. 1 ▸*b*), a well known, characterized protein binding region (Palomo *et al.*, 2011 ▸).

## 2. Methods

### 2.1. KinCoRe

KinCoRe is a web resource which curates protein kinase structures from the PDB and assigns conformational and inhibitor type labels (Modi & Dunbrack, 2022 ▸). We utilized the KinCoRe database with keyword ‘GSK3β’, which yielded 204 total chains, 111 unique PDB entries and 88 distinct co-crystallized ligands (data extracted March 11th, 2026). For each kinase chain, we extracted the KinCoRe annotations for DFG spatial state, DFG dihedral state, salt-bridge state, activation-loop status and phosphorylation status. Structures were assigned to conformational categories according to the unique combinations of these KinCoRe-provided annotations. Several GSK3β PDB entries contained multiple macromolecular chains, resulting in duplicate representations of the same structure. For example, PDB entry 8auz appeared as two entries (8AUZA and 8AUZB), corresponding to distinct chains. To eliminate structural redundancy, duplicates were manually inspected for redundancy and removed. A single representative chain (chain *A*) was retained for each PDB entry. For consistency, chain identifiers were omitted during labeling; thus, retained entries are referenced by PDB code only (for example 8auz). Chains which have any motif residue missing or do not satisfy the dihedral distance cutoff criteria as defined by Modi and Dunbrack are assigned the dihedral label ‘None’ (Modi & Dunbrack, 2019 ▸, 2022 ▸). PDB entries 8qjiand 4dit are assigned as ‘None’ in the KinCoRe dataset and were excluded from our structural analysis. The focus of this comprehensive analysis is on experimentally determined structures. *AlphaFold*-derived models included in the KinCoRe dataset (AF-P49841-K1A and AF-P49841-K3A) were excluded. The complete dataset, along with detailed inclusions and exclusions criteria, is provided in Supplementary Table S1 and the Supplementary Data. The kinase conformational states identified in this study are labeled in accordance with kinase nomenclature as described previously (Modi & Dunbrack, 2019 ▸).

### 2.2. PyMOL

Structural superimpositions, αC–β5 loop configurations and visualizations were performed in *PyMOL* (version 3.1.8; Schrödinger, New York, USA). Representative structures from each conformational cluster were aligned, and inlay views of the salt bridge, C-terminal activation loop, DFG motif, αC–β5 loop and Tyr216 phosphorylation state were generated to highlight structural differences. Local root-mean-square deviations (RMSDs) were calculated across backbone α carbons (C^α^) of the αC–β5 loop (residues 89–94) after global structural alignment using the *CE* algorithm in *PyMOL* (Shindyalov & Bourne, 1998 ▸). The localized displacement of the loop was quantified by calculating the 3D displacement vector between the geometric centroids of residues 89–94 by applying an established ligand-induced displacement analysis (van Westen *et al.*, 2010 ▸). The obtained centroid displacement distances were sorted into three classes: small (≤2 Å), medium (between 2 and 4 Å) and large (≥4 Å) as described previously (van Westen *et al.*, 2010 ▸). Accordingly, we classified a displacement distance of ≥4 Å to represent the αC–β5 loop-out conformation.

## 3. Results and discussion

### 3.1. Historical landscape of deposited GSK3β X-ray crystal structures

KinCoRe data extraction identified 204 GSK3β chains corresponding to 111 unique deposited Protein Data Bank (PDB) structures deposited between 2002 and 2025. All GSK3β structures were resolved by X-ray crystallography. The 111 deposited structures include 88 unique ligands co-crystallized with GSK3β classified as ATP-like, Type I, Type I^1/2^ or peptide, and structural resolutions range from 1.39 to 3.5 Å (Fig. 2 ▸). The highest resolution crystal structure of GSK3β, 1.39 Å, was published in 2026 (PDB entry 9x2v; Haslböck *et al.*, 2026 ▸). The landmark crystal structures of GSK3β were reported in 2001 (PDB entries 1i09, 1h8f and 1gng; Bax *et al.*, 2001 ▸; Dajani *et al.*, 2001 ▸; ter Haar *et al.*, 2001 ▸). The first two deposited structures represent apo human GSK3β (Dajani *et al.*, 2001 ▸; ter Haar *et al.*, 2001 ▸), whereas the third contains GSK3β in complex with the FRATtide peptide (Bax *et al.*, 2001 ▸). Structures of GSK3β in complex with AMPNP and ADP were subsequently reported in 2004 (PDB entries 1j1b and 1j1c, respectively; Aoki *et al.*, 2004 ▸). Early landmark structures of GSK3β in complex with small-molecule inhibitors included a complex with the ADZ ligand (PDB entry 1o9u; Dajani et al., 2003 ▸), as well as complexes with stauro­sporine, indirubin-3′-monoxime and inhibitor I-5 (PDB entries 1q3d, 1q41 and 1q4l, respectively; Bertrand *et al.*, 2003 ▸). A structure of GSK3β in complex with the ATP-competitive inhibitor AR-A014418 was also reported (PDB entry 1q5k; Bhat *et al.*, 2003 ▸). Together, these early studies established a structural foundation for understanding GSK3β ligand recognition and regulation, providing a framework for subsequent analyses of its conformational diversity. A detailed taxonomic analysis of this dataset is available in Supplementary Fig. S1. Briefly, GSK3β has been structurally resolved across three distinct species: *Homo sapiens*, *Mus musculus* and *Toxoplasma gondii*.

### 3.2. The GSK3β ligand landscape reveals the five most prevalent co-crystallized ligands

Comprehensive analysis of all deposited GSK3β PDB structures reveals that the kinase has been co-crystallized with four primary ligand classes: ATP-like (four structures), Type I (88 structures), Type I^1/2^ (one structure) and peptide ligands (16 structures) (Fig. 2 ▸*a*). GSK3β is most frequently observed in complex with Type I inhibitors and peptide ligands, which together account for the majority of available structures. After manual review of the KinCoRe dataset, we identified two structures that are in the apo condition or bound to ‘no ligand’ (PDB entries 1i09 and 1h8f). The two structures lacking a bound ligand were included in the subsequent conformational classifications.

Notably, no Type III (allosteric) small-molecule inhibitors have been co-crystallized with GSK3β to date. Although KinCoRe annotates ligand PG6 (PDB entry 9x2v) as allo­steric, the ligand corresponds to a high-affinity peptide inhibitor (BiS3, *K*_d_ = 0.3 n*M*; Haslböck *et al.*, 2026 ▸). Accordingly, we classify PG6 as a peptide ligand in this study.

The resolution of GSK3β structures varies by ligand class, ranging from 1.8 to 2.6 Å for ATP-like complexes, 1.85 to 3.5 Å for Type I inhibitors, 3.2 Å for the single Type I^1/2^ structure (excluded from resolution-range comparisons) and 1.39 to 2.6 Å for peptide-bound structures (Fig. 2 ▸*b*). Across all structures, the BLA-minus conformation predominates (Fig. 2 ▸*c*), with only two structures adopting the ABA-minus conformation: one in complex with a Type I inhibitor and one with a peptide ligand.

To further characterize ligand prevalence, we identified the most frequently observed individual co-crystallized ligands. The chemical structures of these five ligands, two ATP-like ligands (ADP and ANP) and three Type I inhibitors (A1IYF, 3NG and 65C), are provided in Supplementary Fig. S2, highlighting the chemical diversity of the dominant ligand classes within the GSK3β structural dataset. ADP and ANP, both ATP-like ligands, are the most common, appearing in nine and three PDB entries, respectively (Table 1 ▸). The next most frequent ligands, A1IYF, 3NG and 65C, are Type I inhibitors, each observed in two independent structures. A summary of ligand identities, classifications, frequencies and corresponding PDB codes is provided in Table 1 ▸.

### 3.3. Applying the KinCoRe structural classification to identify conformational states of GSK3β

We applied KinCoRe’s established structural classification clustering framework based on DFG spatial geometry and DFG backbone/side-chain dihedrals, with defined reference clusters and exclusion criteria, to the GSK3β crystallographic structural dataset (Gizzio *et al.*, 2026 ▸; Modi & Dunbrack, 2019 ▸, 2022 ▸). This nomenclature convention distinguishes between kinase activity-like states, where each kinase is grouped into three spatial groups (DFG-in, DFG-out or DFG-inter) based on the positioning of the DFG-Phe side chain (Modi & Dunbrack, 2019 ▸). All GSK3β experimentally validated structures are conformationally in the DFG-in spatial group. Within these groups, KinCore further clusters by the dihedral angles positioning the DFG-Phe side chain and names according to the region occupied by the angles on the Ramanchandran map (‘A’ for alpha, ‘B’ for beta, ‘L’ for left-handedness) and the Phe side-chain rotamer (‘plus’ for +60°, ‘minus’ for −60° or ‘trans’ for 180°; Modi & Dunbrack, 2019 ▸, 2022 ▸). The dihedral labels are distinguished based on residues in the XDFG sequence of kinases (Modi & Dunbrack, 2019 ▸). GSK3β exists in two major DFG-in clusters: the BLA-minus and ABA-minus states (a total of 107 BLA-minus structures and two ABA-minus structures). The DFG-in clusters, BLA-minus (Fig. 3 ▸*a*) and ABA-minus (Fig. 3 ▸*b*), differ in the positional shift of the cysteine residue flanking the DFG motif (Fig. 3 ▸). This results in variations of active-like and inactive-like kinase states observed in deposited GSK3β structures. While BLA-minus broadly represents the active-like state of kinases, certain well established features within a BLA-minus state such as salt-bridge orientation and activation-loop positioning can influence kinase activity status. Here, we also include an additional feature, the αC–β5 loop, which has been mentioned in prior publications but not included in a conformational analysis of GSK3β (Stamos *et al.*, 2014 ▸). The chains that have any motif residue missing or do not satisfy the dihedral distance cutoff criteria are assigned the dihedral label ‘None’ by KinCoRe clustering (Modi & Dunbrack, 2019 ▸, 2022 ▸). In our dataset, there are two structures assigned as ‘None’ (PDB entries 8qji and 4dit), which were excluded from our conformational analysis. The complete conformational ensemble of existing X-ray crystal structures of GSK3β is summarized in Table 2 ▸.

### 3.4. Classification of GSK3β conformations and activity-like states

Firstly, to evaluate the catalytic salt-bridge state in our structures, we considered both the 3.6 Å boundary utilized by KinCoRe and a stricter 3.3 Å limit noted in the literature (Karshikoff & Jelesarov, 2008 ▸; Modi & Dunbrack, 2022 ▸). The 3.2–3.3 Å range represents a highly optimized hydrogen bond required for maximum atomic orbital overlap between the N atom of the conserved lysine residue (Lys N^ζ^) and the O atom of the conserved glutamate residue (Glu O^ɛ^) (Karshikoff & Jelesarov, 2008 ▸; Sanchez *et al.*, 2026 ▸). A single GSK3β structure possesses a disrupted catalytic salt-bridge interaction (Saltbr-out) with a distance of 3.66 Å. While this places it just past the standard KinCoRe 3.6 Å cutoff and well beyond the highly optimized 3.3 Å threshold, referencing both metrics clarifies that our structure captures the salt bridge at the extreme boundary of a disrupted state.

Next, active kinases require both an intact catalytic salt bridge and an extended activation loop for catalysis, and are defined here with Saltbr-in and ActLoop-in configurations, respectively. KinCoRe classifies an extended activation loop or ActLoop-in based on two criteria: an N-terminal DFG backbone hydrogen-bond distance of less than 3.6 Å (ActLoopNT-in) and C-terminal APE motif dihedral and distance constraints (ActLoopCT-in) (Gizzio *et al.*, 2026 ▸; Modi & Dunbrack, 2019 ▸). While all deposited GSK3β structures satisfy the ActLoopNT-in condition, variation in ActLoopCT determines whether the overall conformation is classified as ActLoop-in or ActLoop-out, and thus stabilizing active-like or inactive-like states. Accordingly, subsequent analyses focus on the variations in ActLoopCT, which is used to assign activation-loop state throughout this study. Notably, an HRD-in configuration is conserved across all GSK3β crystal structures and was not included further in our discussion. Lastly, we included the presence of the regulatory phosphorylated Tyr216 in our structural analysis (Buescher & Phiel, 2010 ▸; Hughes *et al.*, 1993 ▸).

Based on the various KinCoRe configurational labels discussed above, we initially identified seven distinct conformations of GSK3β. However, by incorporating the structural plasticity of the αC–β5 loop (residues 89–94), we expanded this conformational landscape to reveal an additional state, bringing the total to eight conformations (Table 2 ▸). Intrigued by previous work that noted substrate specificity via conformational changes on this region (Stamos *et al.*, 2014 ▸), we sought to consider the plasticity of the αC–β5 loop in the GSK3β conformational landscape. The αC–β5 loop is also referred to as the C loop in some GSK3β publications to denote the linker between β5 and the αC helix and should not be confused with the catalytic C loop. For consistency with kinase naming conventions, here we have used ‘αC–β5 loop’ to describe the loop between β5 and the αC helix (Fig. 1 ▸*c*). Using a ligand-induced displacement analysis, we calculated αC–β5 loop centroid displacement (van Westen *et al.*, 2010 ▸). As previously described, average centroid displacement distances that are greater than equal to 4 Å are considered to be large displacements (van Westen *et al.*, 2010 ▸). Through loop centroid displacement comparisons across GSK3β structures, we discovered that seven GSK3β structures have large, localized displacement of the αC–β5 loop (αC–β5-out; Supplementary Table S2). This provides novel insight into the GSK3β conformational landscape, particularly for researchers interested in exploiting αC–β5-loop plasticity and for the development of conformationally selective inhibitors.

Through this additional classification, we identified a total of eight conformational states stabilized by GSK3β in crystal structures (Table 2 ▸). Supplementary Table S3 provides the observed conformational frequencies for GSK3β. The most frequent combination occurred in 51 structures (Conformation 1, 46.8%), followed by combinations observed in 25 structures (Conformation 3, 22.9%), 13 structures (Conformation 6, 11.9%), ten structures (Conformation 5, 9.2%) and seven structures (Conformation 2, 6.4%). Three combinations were each represented by a single structure (Conformations 4, 7 and 8; 0.9% each) and therefore their prevalence and biological recurrence cannot yet be established. Detailed conformational assignments for each evaluated GSK3β PDB entry are provided in the supporting information.

### 3.5. Structural landscape of the eight stabilized GSK3β conformations

We next examined each GSK3β conformational state individually to characterize their structural features and assess their potential functional implications. Supplementary Fig. S3 presents an overlay of representative structures, one for each conformational state listed in Table 2 ▸. Fig. 4 ▸ compares selected structural features of these states with those of Conformation 1, which serves as a reference structure (Fig. 4 ▸*a*), highlighting the differences in key regulatory regions.

#### 3.5.1. BLA-minus/Saltbr-in/ActLoop-in (active-like state)

Structures in this state adopt three unique canonical active kinase conformations (Conformations 1, 2 and 3), characterized by a BLA-minus, DFG-in arrangement, an intact catalytic salt bridge (Saltbr-in) and an extended activation loop (ActLoop-in), together with the conserved HRD-in motif (representative PDB entries 2jld, 8vmf and 5oy4; Table 2 ▸). The activation loop satisfies both ActLoopNT-in and ActLoopCT-in criteria, and the Lys85–Glu97 interaction of ≤3.6 Å is maintained, supporting predicted catalytic competence (2.9 Å, PDB entry 2jld).

While all three representative structures, PDB entries 2jld, 8vmf and 5oy4, adopt the same configuration of regulatory regions, there is a notable shift in the αC–β5 loop and variation in the Tyr216 phosphorylation state. The 8vmf structure, which is bound to a β-catenin S45D peptide ligand, contains an extended αC–β5 linker with the Phe93 residue tilted at the top of the loop (Fig. 4 ▸*b*). The Phe93 residue has previously been noted to confer substrate specificity via conformational changes upon substrate binding and to move 8.5 Å (Stamos *et al.*, 2014 ▸). Additionally, the 5oy4 structure contains a phosphorylated activating tyrosine residue in the activation loop, pTyr216 (Dajani *et al.*, 2003 ▸; Hughes *et al.*, 1993 ▸), making it distinct from PDB entries 2jld and 8vmf (Fig. 4 ▸*c*). These subtle conformational differences can be key for choosing a suitable structure for experimental studies and avoiding false negatives during computational drug/ligand screening. Overall, these combined features collectively position key residues for substrate binding and phosphoryl transfer, consistent with an active-like kinase state.

#### 3.5.2. BLA-minus/Saltbr-out/ActLoop-in (inactive-like state)

Although these structures retain a BLA-minus DFG-in conformation and extended activation loop, the disruption of the salt bridge (Saltbr-out) renders these conformations incompatible with catalysis (PDB entry 9huk; Fig. 4 ▸*d*). In PDB entry 9huk, the Lys85–Glu97 bond distance is greater than 3.6 Å, leading to the inability to form the key interaction, resulting in a Saltbr-out conformation (3.66 Å, Fig. 4 ▸*d*).

#### 3.5.3. BLA-minus/Saltbr-in/ActLoop-out (inactive-like state)

These GSK3β structures maintain a BLA-minus DFG-in configuration and an intact catalytic salt bridge while the activation loop adopts a collapsed (ActLoop-out) conformation as shown in PDB entries 4nm0 and 1o9u (Fig. 4 ▸*e*). Despite the alignment of key catalytic residues, this spatial rearrangement occludes substrate accessibility, resulting in an inactive-like kinase state.

PDB entry 1o9u is distinct from 4nm0 due to a phosphorylated tyrosine residue in the activation loop, pTyr216 (Fig. 4 ▸*f*). While N-terminal phosphorylation at Ser9 is known to functionally override GSK3β activation, whether PDB entry 1o9u features a pSer9 remains unconfirmed (Bhat *et al.*, 2000 ▸). Since the resolution of the N-terminus is limited in this structure, a common constraint across several GSK3β structures, we deliberately avoid overinterpreting these structural data. These localized N-terminal difference or varying phosphorylation profiles lead to a structural divergence between PDB entries 4nm0 and 1o9u within the same conformational subcategory (BLA-minus/Saltbr-in/ActLoop-out).

#### 3.5.4. ABA-minus/Saltbr-in/ActLoop-in (inactive-like state)

These structures exhibit an ABA-minus DFG-in configuration with an intact salt bridge and extended activation loop (PDB entry 5air; Fig. 4 ▸*g*). Despite satisfying several canonical active-like state features, the altered DFG geometry and associated backbone rearrangements, particularly near the cysteine flanking the DFG motif, perturb ATP or substrate positioning, and these conformations are annotated as inactive.

#### 3.5.5. ABA-minus/Saltbr-in/ActLoop-out (inactive-like state)

Structures in this group display multiple inactivating features, including an ABA-minus configuration and a collapsed activation loop (ActLoop-out), with a phosphorylated tyrosine residue, pTyr216 (PDB entry 6y9s; Fig. 4 ▸*h*). The combined loss of catalytic residue coordination and activation-loop extension renders these conformations incompatible with catalysis, consistent with the stabilized inactive-like states observed in crystallographic datasets.

## 4. Discussion

Multiple studies have shown that kinase structural flexibility, the availability of multiple PDB structures and the selection of target conformations can substantially dictate molecular-docking performance, predicted binding modes and ligand-ranking accuracy (Lake *et al.*, 2018 ▸; Schaller *et al.*, 2024 ▸; Suri *et al.*, 2026 ▸). In particular, successful docking at allosteric sites may depend on the availability of a target conformation that accommodates the relevant regulatory pocket and ligand-binding geometry (Chen *et al.*, 2026 ▸). Therefore, the conformational diversity observed among GSK3β structures should be viewed not only as a source of uncertainty for computational modeling, but also as an opportunity for selective inhibitor discovery. A distinct GSK3β conformation may expose or stabilize structural features that can be exploited by ligands designed to achieve greater selectivity than conventional ATP-competitive inhibitors.

An ideal GSK3β inhibitor would achieve disease-relevant modulation while minimizing disruption of the homeostatic catalytic functions of GSK3β. In the past, GSK3β inhibition has been explored as a therapeutic strategy for type 2 diabetes, as kinase inhibition can improve blood glucose handling. However, due to the roles of GSK3β in cardiovascular hypertrophic signaling, the chronic inhibition of GSK3β has been associated with myocardial hypertrophy in experimental settings (Huisamen *et al.*, 2016 ▸). This illustrates the challenge of broadly inhibiting a constitutively active and widely expressed kinase. It also supports the development of biologically selective therapeutic strategies that target potential noncatalytic sites or noncanonical functions of GSK3β (Singh *et al.*, 2026 ▸).

Alternative approaches may include conformation-selective inhibitors, allosteric ligands, protein–protein interaction inhibitors and targeted protein-degradation strategies such as proteolysis-targeting chimeras (PROTACs). These approaches may improve selectivity through several mechanisms. A conformation-selective or allosteric inhibitor could preferentially engage a disease-associated structural state or regulatory pocket. A protein–protein interaction inhibitor could disrupt a disease-relevant scaffolding or substrate-recruitment function while preserving a proportion of the catalytic activity of GSK3β. Similarly, a targeted degrader may enable selective intervention in biologically defined cellular contexts, although its therapeutic value would depend on tissue exposure, degradation selectivity and the consequences of reducing total GSK3β protein abundance. Collectively, these strategies may reduce the systemic toxicity associated with broad and continuous ATP-site inhibition.

Since GSK3β is a promiscuous kinase with extensive substrate overlap across disease contexts, conformation-selective inhibitors offer a route to context-specific pharmacology. The present study identifies eight crystallographically observed GSK3β conformations, including distinct combinations of DFG dihedral state, catalytic salt-bridge status, activation-loop orientation, αC–β5-loop configuration and phosphorylation state. Recent work examining the effect of protein conformational state on docking performance illustrates why structure selection requires target-specific evaluation. Suri *et al.* (2026 ▸) compared apo and holo structures in redocking and cross-docking workflows for GSK3β and β-site amyloid precursor protein-cleaving enzyme 1 (BACE1; Suri *et al.*, 2026 ▸). For GSK3β, the apo structure reportedly showed greater docking selectivity than the holo structure, whereas the holo BACE1 structure provided superior pose recovery (Suri *et al.*, 2026 ▸). These contrasting observations demonstrate that no universal preference for apo or holo structures can be assumed across targets. Instead, docking performance depends on the relationship between the target conformation, the query-ligand chemotype, the binding site and the intended application. For GSK3β, this supports the use of multiple representative conformations or ligand-bound states rather than reliance on a single deposited structure.

Similarly, another group used available GSK3β X-ray crystal structures in computational docking studies and identified GSK3β residues that contribute to induced-fit inter­actions (Boesger *et al.*, 2025 ▸). This group acknowledged the plethora of and variability among the deposited crystal structures and chose their structures based on local GSK3β residues (Boesger *et al.*, 2025 ▸). When the three highlighted PDB structures from that study were mapped onto the present classification framework, all were assigned to Conformation 1. This state is characterized by a BLA-minus dihedral configuration, an intact salt bridge and an extended activation loop. Therefore, we speculate that Conformation 1 may serve as a useful reference state for modeling ligands expected to bind the canonical ATP site within an active-like GSK3β architecture. However, its high frequency in deposited structures should not be interpreted as evidence that it is universally optimal for docking campaigns, inhibitor classes or certain disease contexts.

PDB entry 1i09 represents an unphosphorylated apo GSK3β structure and may be a useful reference or control for docking studies designed to assess ligand compatibility with an apo-like kinase architecture. However, it should not be presumed to be universally optimal. We recommend including PDB entry 1i09 alongside ligand-bound representative structures from the same and alternative conformational categories, followed by target- and chemotype-specific validation through redocking, cross-docking, enrichment analysis and, where possible, experimental testing.

Less frequently observed states with disrupted catalytic salt bridges or altered activation-loop orientations may be especially relevant to induced-fit and conformation-selective binding mechanisms. Although these categories are represented by relatively few deposited structures, they may expose local interaction opportunities that are not present in the predominant active-like state. For example, conformational variation surrounding the residue preceding the DFG motif, Cys199, may affect the geometry and accessibility of the ATP-binding region. Cys199 has been implicated in GSK3β–inhibitor interactions, including hydrogen-bonding contacts involving its thiol group (Boesger *et al.*, 2025 ▸; Ghazanfari *et al.*, 2021 ▸). Similarly, the less frequently observed ABA-minus states may provide distinct local structural environments that warrant further investigation as potential templates for selective ligand design.

Ultimately, a more complete understanding of the relationship among ligand chemistry, target conformation, inferred catalytic competence and cellular phenotype may enable the development of more selective and biologically informed GSK3β therapeutic strategies.

## 5. Conclusion and future directions

This study defines the experimentally observed conformational landscape of GSK3β from 111 experimental X-ray crystal structures, spanning three species, with reported resolutions ranging from 1.39 to 3.5 Å and 88 unique co-crystallized ligands. Across this dataset, all GSK3β structures adopt a DFG-in configuration and are associated with ATP-like ligands, Type I inhibitors, Type I^1/2^ inhibitors or peptides. The structures occupy either BLA-minus or ABA-minus dihedral states. ATP-like ligands, including ATP and ADP, and Type I inhibitors were the most frequently represented ligand classes. Importantly, the GSK3β dataset lacks experimentally validated structures in a DFG-out state or in complex with a Type III inhibitor, highlighting a substantial gap in the structural characterization of noncanonical or allosteric inhibitory mechanisms.

Among the 109 analyzed GSK3β structures included in our conformation analysis, eight unique combinations of structural annotations were observed. For each kinase chain, the KinCoRe-derived DFG spatial and dihedral classifications, salt-bridge status, activation-loop annotation, phosphorylation status and αC–β5-loop configuration were integrated into a deterministic conformational state key. The most prevalent state, Conformation 1, accounted for 51 structures (46.8%), followed by Conformation 3 with 25 structures (22.9%) and Conformation 6 with 13 structures (11.9%) structures. Three conformations were represented by one structure each (Conformations 4, 7 and 8; 0.9% each). These states were retained as distinct conformations based on their unique structural annotation profiles; however, their single-structure representation indicates that they are presently rare or under-sampled within the available GSK3β crystallographic dataset. Low-frequency categories, including those represented by PDB entries 9huk, 5air or 6y9s, should be regarded as provisional structural observations rather than established recurrent GSK3β conformational states. Additional structures, ideally obtained across independent constructs, ligands, crystallization conditions and complementary solution-based experiments, will be required to determine whether these profiles reflect rare biologically relevant conformations, ligand- or construct-dependent states, crystal-packing effects or incomplete sampling of the GSK3β conformational landscape.

Canonical kinase activity is commonly inferred from features such as a DFG-in motif, BLA-minus dihedral state, HRD-in configuration and intact catalytic salt bridge (Modi & Dunbrack, 2022 ▸). However, our analysis demonstrates that additional criteria can be incorporated to capture the conformational diversity of GSK3β. Incorporating the C-terminal activation-loop orientations (Gizzio *et al.*, 2026 ▸) and αC–β5-loop configuration with Tyr216 phosphorylation state refined the classification of GSK3β structures and sheds light on why certain globally DFG-in configurations stabilize inactive-like features. In particular, regardless of whether the kinas adopted a BLA-minus or ABA-minus dihedral label, structures with an ActLoop-out were classified as inactive-like. These findings emphasize that global DFG state should not be interpreted in isolation when evaluating GSK3β inferred catalytic competence or selecting structural models for virtual screening, hit validation and protein–ligand binding analyses.

The analysis further revealed that chemically distinct Type I inhibitors can stabilize distinct GSK3β conformations and activity-like structural states. Four representative structures, PDB entries 2jld, 9huk, 1o9u and 6y9s, contain the Type I inhibitors AG1, A1IXL, ADZ and OHK, respectively, and represent unique four conformational states (Table 2 ▸, Supplementary Fig. S4). These ligands differ in their core chemical scaffolds and their complexes exhibit distinct combinations of activation-loop configuration, catalytic salt-bridge configuration and Tyr216 phosphorylation state (Supplementary Table S4).

Conformation 8 (representative PDB entry 6y9s) exhibits an inactive-like state characterized by a ABA-minus state, activation-loop displacement, an intact catalytic salt bridge and Tyr216 phosphorylation. Its bound ligand, OHK, contains an imidazo[1,5-a]pyridine-3-carboxamide (IMID 2), comprising a fused imidazole-pyridine ring with a carboxamide substituent (Supplementary Fig. S4, Supplementary Table S4). In contrast, Conformation 4 (representative PDB entry 9huk) adopts a BLA-minus state with a disrupted catalytic salt bridge and an extended activation loop. The bound inhibitor, A1IXL, contains a 2-oxobenzimidazoline scaffold, comprising a benzene ring fused to a five-membered ring containing two N atoms and a carbonyl group (Supplementary Fig. S4). Conformation 1 (representative PDB entry 2jld), bound to the organoruthenium half-sandwich inhibitor AG1, adopts an active-like conformation, with an intact salt bridge and extended activation loop, despite the absence of activation phosphorylation (Supplementary Fig. S4). Finally, Conformation 6 (representative PDB entry 1o9u), bound to the minimal purine inhibitor ADZ, exhibits a BLA-minus state with activation-loop displacement and Tyr216 phosphorylation (Supplementary Fig. S4). Together, these examples demonstrate that Type I inhibitor binding is compatible with multiple GSK3β conformations rather than a single uniform structural state (Supplementary Fig. S4). The observed variation across chemically distinct inhibitor complexes underscores the importance of considering activation-loop geometry, salt-bridge integrity and phosphorylation status alongside ligand-binding classification when interpreting the structural consequences of GSK3β inhibition.

This conformational diversity has practical implications for structure-based drug discovery. ATP-competitive ligands may engage the hinge region, catalytic lysine, hydrophobic subpockets and residues such as Cys199 through distinct combinations of hydrogen bonding, van der Waals interactions and hydrophobic contacts (Boesger *et al.*, 2025 ▸; Ghazanfari *et al.*, 2021 ▸). These differences in ligand–protein interactions provide a potential basis for the distinct conformational states observed among inhibitors with the same broad binding mechanism. The structural diversity identified here therefore supports the potential of repurposing existing GSK3β inhibitors for conformation-selective inhibitor development and reinforces the value of systematically cataloging kinase conformational states.

For molecular docking, virtual screening, hit validation and protein–ligand binding analyses, the choice of GSK3β template structure should therefore be guided by more than crystallographic resolution, ligand identity or broad DFG classification. Selection should account for (i) global kinase conformation, (ii) local regulatory-element features, including activation-loop orientation, salt-bridge status, αC–β5-loop configuration and phosphorylation state, and (iii) the predicted catalytic competence of the selected structure. Using a structurally incompatible target conformation may reduce docking accuracy, obscure favorable ligand inter­actions and increase the risk of false-negative results during virtual screening and early-stage drug discovery. The conformational assignments reported for each GSK3β PDB entry, provided in the supporting information, offer a practical framework for informed structure selection.

The limitations for the present study should be acknowledged. This study did not perform a systematic chemical fingerprint, scaffold or pharmacophore analysis of co-crystallized GSK3β ligands. Accordingly, the present work identifies associations between ligand-bound structures and conformational states but does not establish which ligand features causally drive a particular GSK3β conformation. Future studies should integrate ligand chemical descriptors, scaffold classifications, binding-region annotations, inhibitory potency values and affinity measurements with the structural state framework presented here. Such analyses could identify molecular features associated with specific conformations and support the rational design of conformation-selective GSK3β inhibitors.

The current dataset also reflects only the conformational diversity captured by available X-ray crystal structures. Additional biologically relevant GSK3β states may exist but remain structurally unresolved, particularly states associated with Type II inhibitors, Type III inhibitors or additional post-translationally modified forms of the kinase. Future structural studies using X-ray crystallography, cryo-electron microscopy, nuclear magnetic resonance spectroscopy and complementary solution-based approaches will be important for expanding the experimentally observed conformational landscape.

In conclusion, this work establishes a structured classification of the currently available GSK3β crystallographic ensemble and identifies eight experimentally observed conformational states defined by integrated global and local structural features. Although future structures may reveal additional states, this framework captures the presently available diversity of GSK3β conformations and provides a foundation for structural interpretation, ligand profiling and conformation-selective drug discovery. More broadly, the findings demonstrate that all DFG-in GSK3β structures are not conformationally equivalent and that comprehensive annotation of regulatory elements is essential for interpreting kinase activity-like states and selecting appropriate structural models for computational and medicinal chemistry applications.

## Supplementary Material

Supplementary Figures and Tables. DOI: 10.1107/S2059798326010533/jb5075sup1.pdf

Supplementary Data. Raw data and GSK3β conformational classifications. DOI: 10.1107/S2059798326010533/jb5075sup2.xlsx

## Figures and Tables

**Figure 1:**
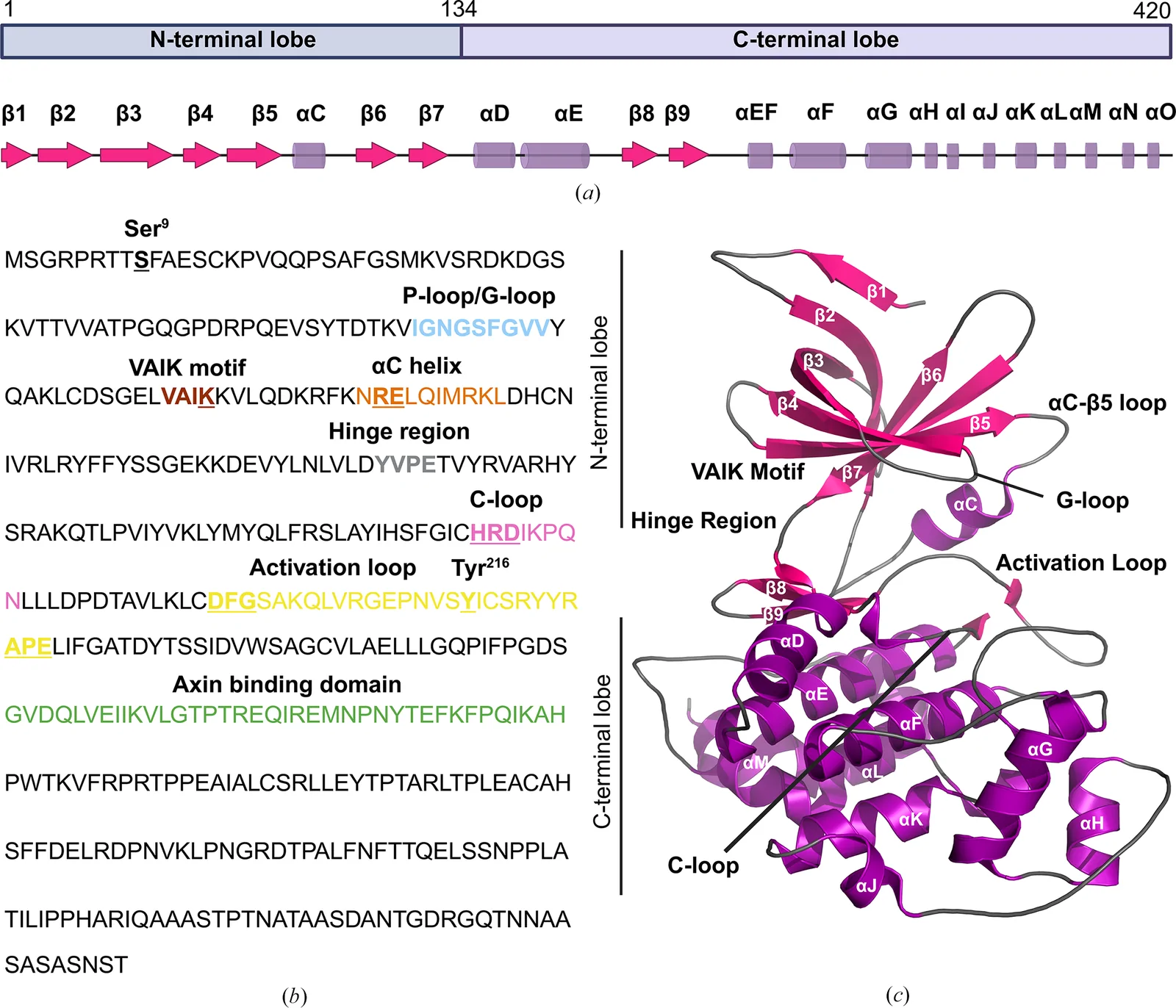
Structural domains and regulatory features of GSK3β. (*a*) Graphical representation of GSK3β domains (top) and secondary-structure elements (bottom). (*b*) Human GSK3β amino-acid sequence (residues 1–420), with key structural and regulatory regions highlighted: the phosphate-binding loop (P-loop), also known as the glycine rich-loop (G-loop; residues 62–70, sky blue), the VAIK motif (residues 82–85; maroon), the αC-helix (residues 95–104, orange), the hinge region (residues 134–137, gray), the catalytic loop (C-loop, 182–186, light pink), the HRD motif (residues 179–181, light pink), the activation loop (residues 200–226, yellow) and the axin-binding domain (ABD, residues 262–299, green). The regulatory residues Ser9, Lys85, Arg96, Glu97, His179, Arg180, Asp181, Asp200, Phe201, Gly202, Tyr216, Ala224, Pro225 and Glu226 are underlined. (*c*) A three-dimensional structure of GSK3β showing various regulatory regions (PDB entry 9huk). The β-strands, α-helices and loops are shown in hot pink, purple and gray, respectively. (*a*) was created in https://BioRender.com.

**Figure 2:**
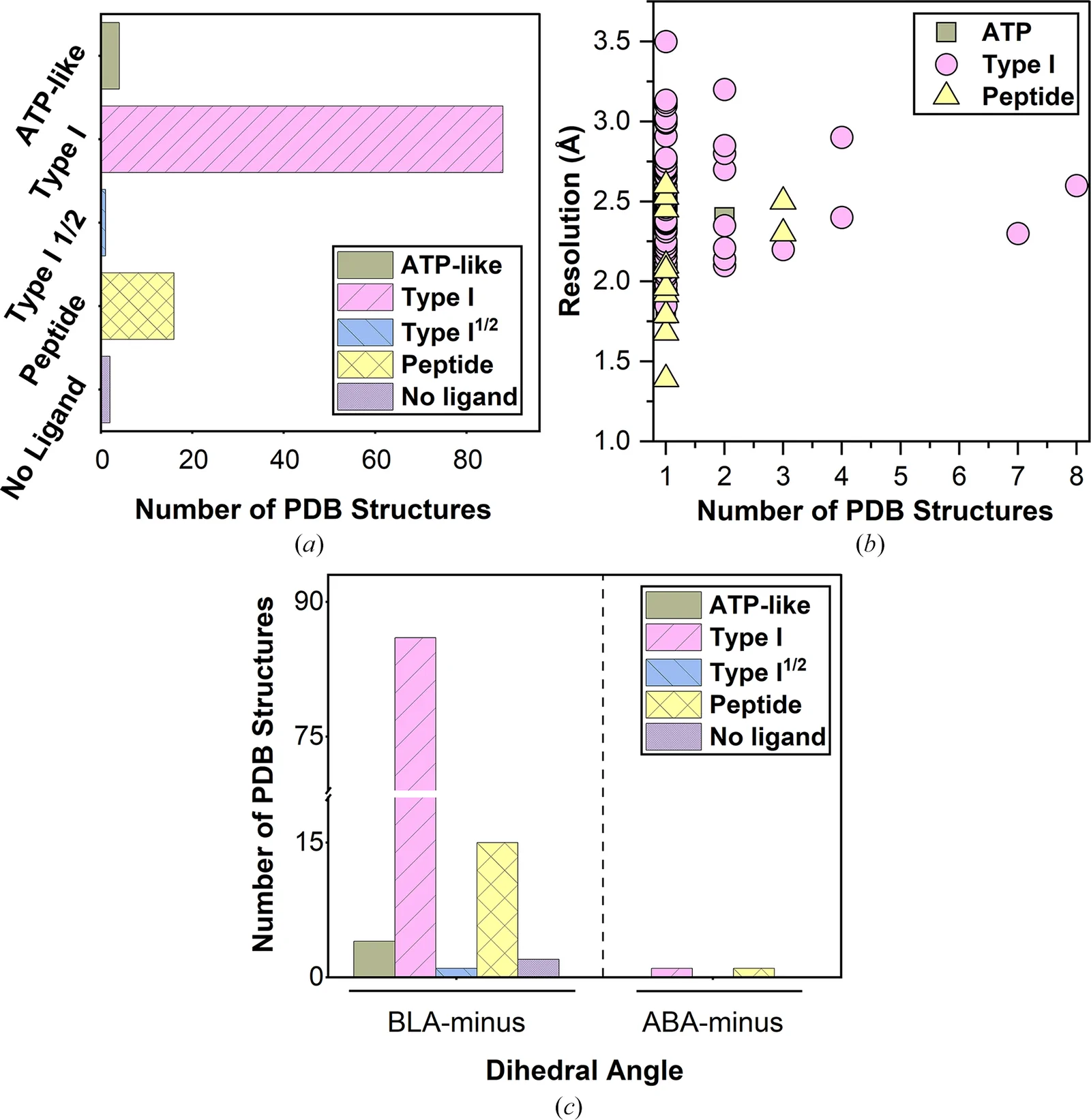
Overview of ligand classes and structural features in co-crystallized structures of GSK3β. (*a*) Frequency of ligand classes in GSK3β structures deposited in the PDB. (*b*) Scatter plot showing the crystallographic resolution (Å) distribution by select co-crystallized ligand classes for 108 GSK3β structures: ATP-like ligand (*n* = 4), Type I ligand (*n* = 88) and peptide (*n* = 16). Apo structures (no ligand, *n* = 2) and structures with Type I^1/2^ ligand (*n* = 1) were excluded from this panel. (*c*) Grouped bar plot highlighting the distribution of ligand classes across different GSK3β dihedral labels.

**Figure 3:**
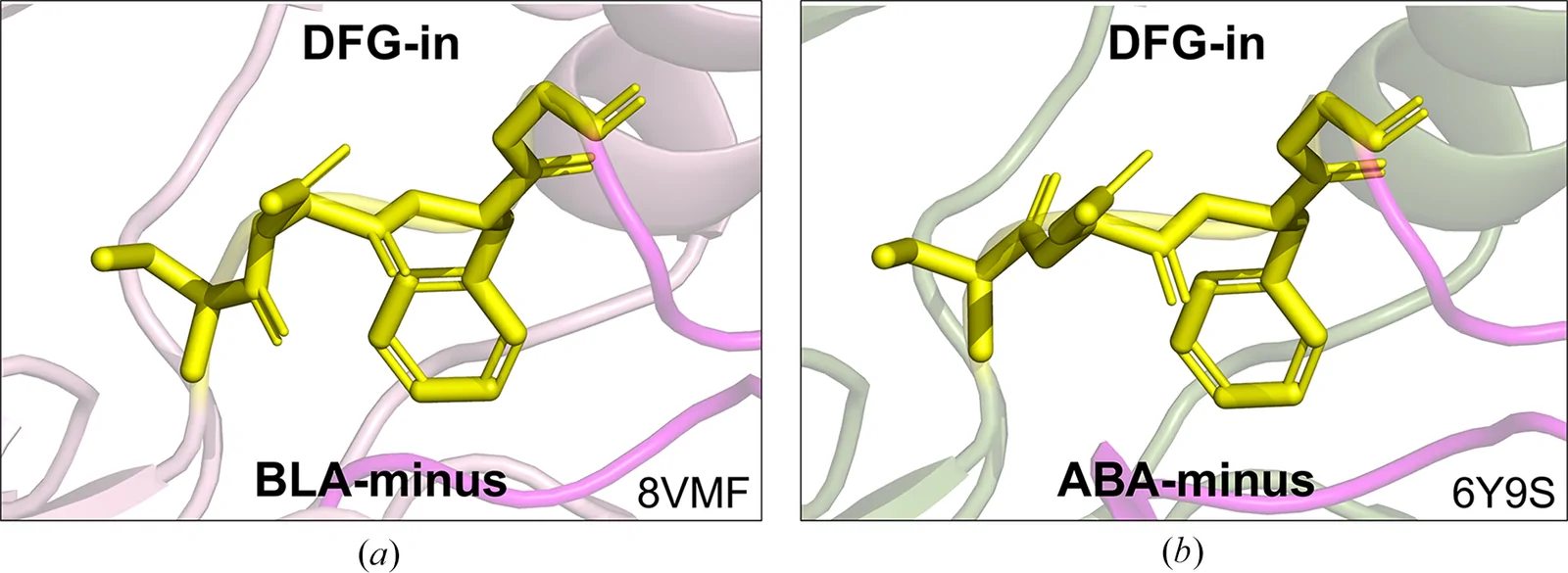
Structural comparison of GSK3β DFG-in conformational clusters. (*a*) GSK3β in the BLA-minus state bound to a β-catenin S45D peptide (Enos *et al.*, 2024 ▸). (*b*) GSK3β in the ABA-minus state bound to the Type I ligand OHK (Buonfiglio *et al.*, 2020 ▸).

**Figure 4:**
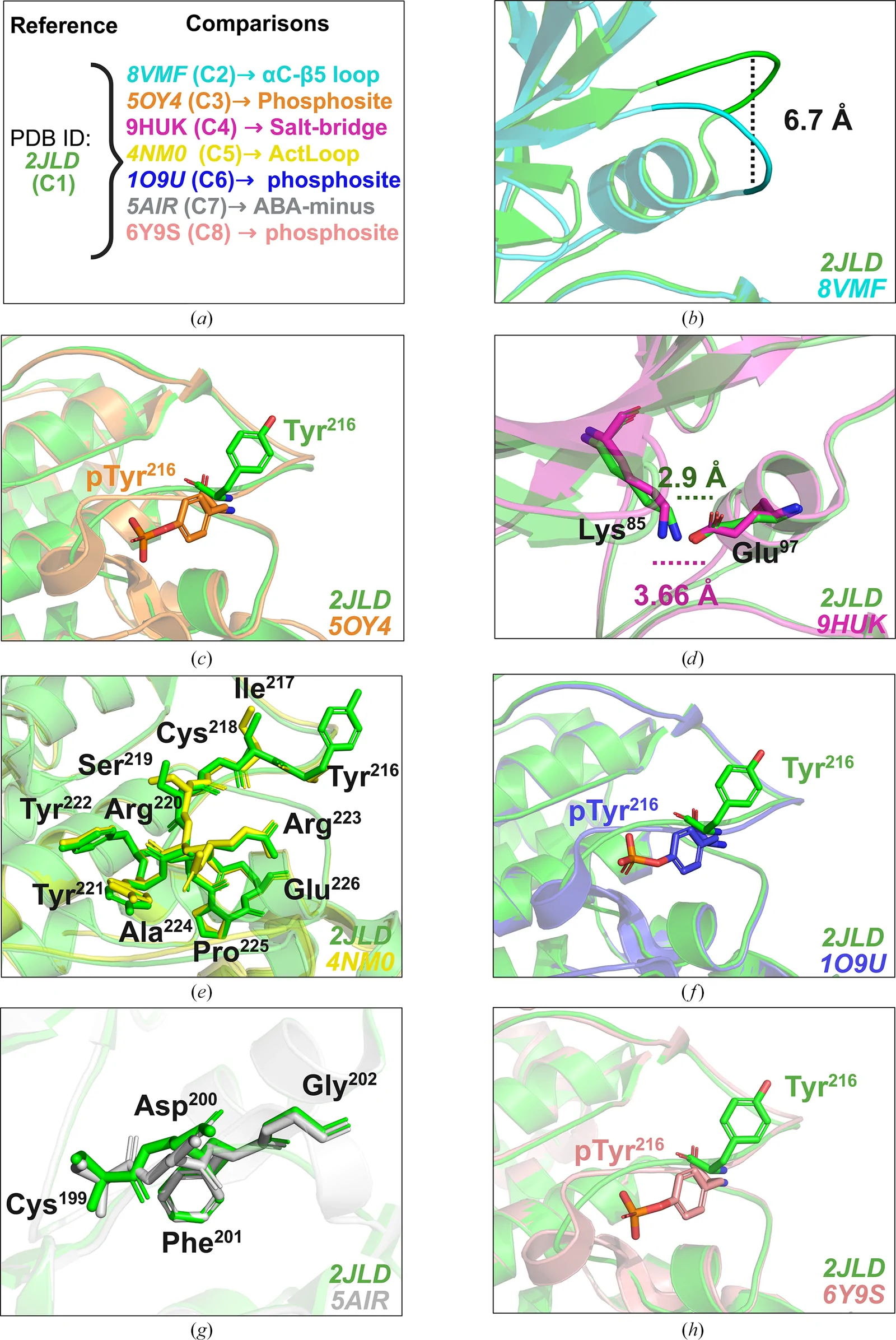
Structural basis of the conformational diversity of GSK3β. (*a*) Overview of the eight GSK3β conformations and the structural features assessed relative to Conformation 1 (reference; representative PDB entry 2jld). Each conformation is represented by a corresponding PDB structure. (*b*) Comparison of the αC–β5-loop configurations. Conformation 1 adopts a αC–β5 loop-in configuration, whereas Conformation 2 (representative PDB entry 8vmf) adopts a αC–β5 loop-out configuration, with a centroid displacement of 6.7 Å relative to Conformation 1. (*c*) Comparison of phosphorylated Tyr216 in Conformation 3 (representative PDB entry 5oy4) with unphosphorylated Tyr216 in Conformation 1. (*d*) Comparison of the intact and displaced Lys85–Glu97 salt-bridge configurations represented by Conformation 1 and Conformation 4 (representative PDB entry 9huk), respectively. The Lys85–Glu97 distance is 2.9 Å in Conformation 1 and 3.66 Å in Conformation 4. (*e*) Comparison of C-terminal activation-loop conformations. Conformation 1 adopts the ActLoop-in configuration, whereas Conformation 5 (representative PDB entry 4nm0) adopts the ActLoop-out configuration. (*f*) Comparison of phosphorylated Tyr216 in Conformation 6 (representative PDB entry 1o9u) with unphosphorylated Tyr216 in Conformation 1. (*g*) Comparison of the DFG-motif dihedral labels. Conformation 1 is assigned the BLA-minus label whereas Conformation 7 (representative PDB entry 5air) is assigned the ABA-minus label. (*h*) Comparison of phosphorylated Tyr216 in Conformation 8 (representative PDB entry 6y9s) with unphosphorylated Tyr216 in Conformation 1.

**Table 1:** Top five co-crystallized GSK3β ligands

Ligand identity	Classification	No. of PDB entries	PDB codes
ADP	ATP-like	9	4nm5, 4nm7, 4nm3, 1j1c, 4nm0, 8vmf, 8vmg, 8vme, 4nu1
ANP	ATP-like	3	1j1b, 8xn6, 1pyx
A1IYF	Type I	2	9hvx, 9hw6
3NG	Type I	2	7z1g, 7z1f
65C	Type I	2	6b8j, 5hln

**Table 2:** Eight unique GSK3β conformations from existing X-ray crystal structure data

Stabilized GSK3β conformation	Representative PDB code	Ligand	Spatial group	Dihedral label	Salt-bridge configuration	Activation-loop configuration	αC–β5-loop configuration	Phosphosite	Inferred catalytic state
1	2jld	Type I	DFG-in	BLA-minus	Saltbr-in	ActLoop-in	αC–β5-in		Active-like
2	8vmf	Peptide	DFG-in	BLA-minus	Saltbr-in	ActLoop-in	αC–β5-out		Active-like
3	5oy4	Type I^1/2^	DFG-in	BLA-minus	Saltbr-in	ActLoop-in	αC–β5-in	Y^216^	Active-like
4	9huk	Type I	DFG-in	BLA-minus	Saltbr-out	ActLoop-in	αC–β5-in		Inactive-like
5	4nm0	Peptide	DFG-in	BLA-minus	Saltbr-in	ActLoop-out	αC–β5-in		Inactive-like
6	1o9u	Type I	DFG-in	BLA-minus	Saltbr-in	ActLoop-out	αC–β5-in	Y^216^	Inactive-like
7	5air	Peptide	DFG-in	ABA-minus	Saltbr-in	ActLoop-in	αC–β5-in		Inactive-like
8	6y9s	Type I	DFG-in	ABA-minus	Saltbr-in	ActLoop-out	αC–β5-in	Y^216^	Inactive-like

## Data Availability

All data are available from the KinCoRe database and Protein Data Bank (PDB). The categorized dataset has been provided in the supporting information.
