# Supplementary material for: Defining the conformational landscape of glycogen synthase kinase 3 beta (GSK3β)
Source: Acta Crystallogr D Struct Biol. 2026 Oct 23;82(Pt 11):1285–97. doi: 10.1107/S2059798326010533 (PMC13717832; doi:10.1107/S2059798326010533)
Supplement: Supplementary file 1 [file d-82-01285-sup1.pdf]

STRUCTURAL BIOLOGY

Volume 82 (2026)

Supporting information for article:

Defining the conformational landscape of glycogen synthase kinase 3 beta (GSK3β)

Akanksha Gurtu and Fernanda Laezza

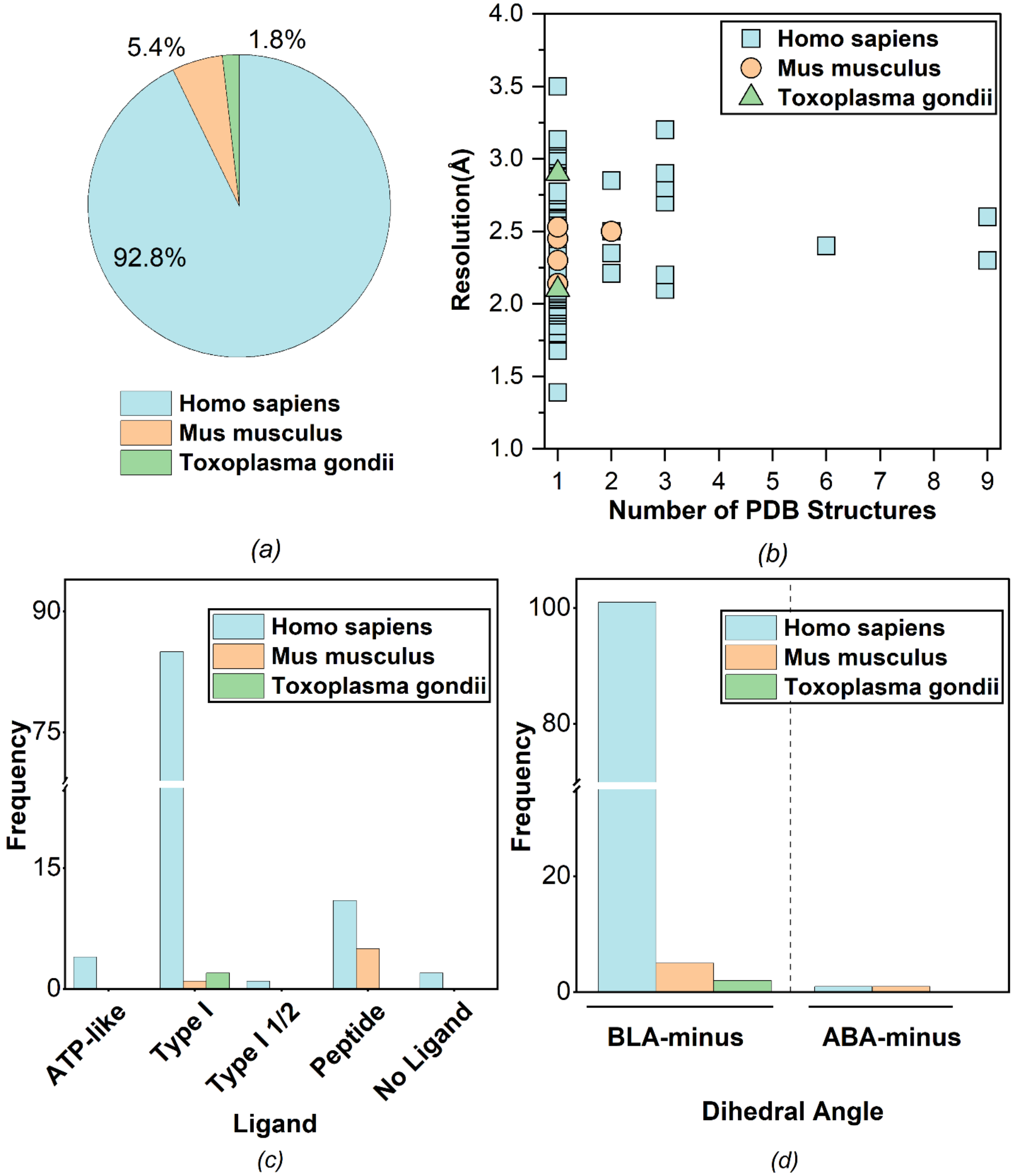

S1. Taxonomic landscape of GSK3β X-ray crystal structures. (a) Taxonomic representation across all deposited GSK3β X-ray crystal structures. (b) Scatter plot representing GSK3β structural resolution (Å) frequency across species (total 111 structures; human n=103 structures, mouse=6 structures,

Toxoplasma gondii=2 structural). (c) Grouped bar plot highlighting the distribution of ligands among species in published PDB structures. (d) Grouped bar plot highlighting GSK3β's conformational profiles across species.

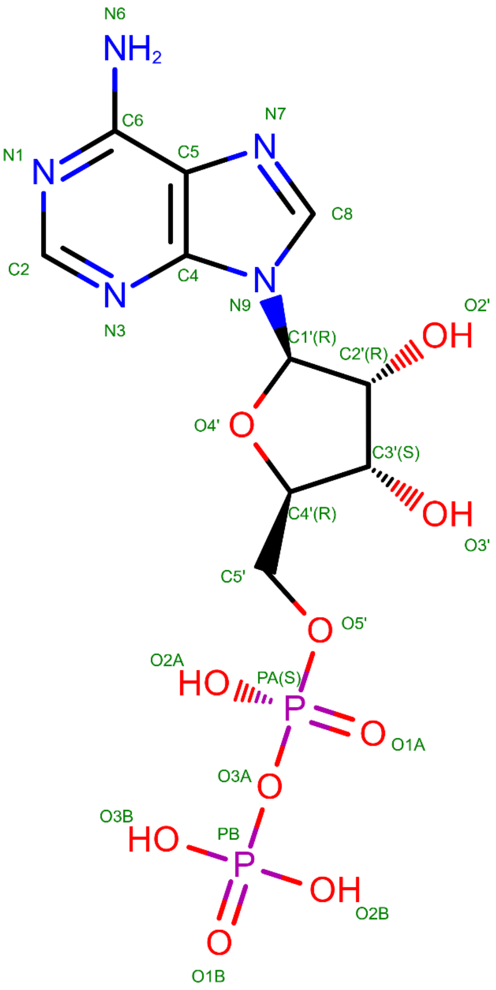
N6
NH2
C6
N7
N1
N
C5
N
C8
C2
C4
N
N
N3
N9
O2'
C1'(R)
OH
C2'(R)
O4'
O
C3'(S)
C4'(R)
OH
O3'
C5'
O
O5'
O2A
HO
PA(S)
P
O
O1A
O3A
O
O3B
HO
PB
P
OH
O2B
O
O1B

ADP

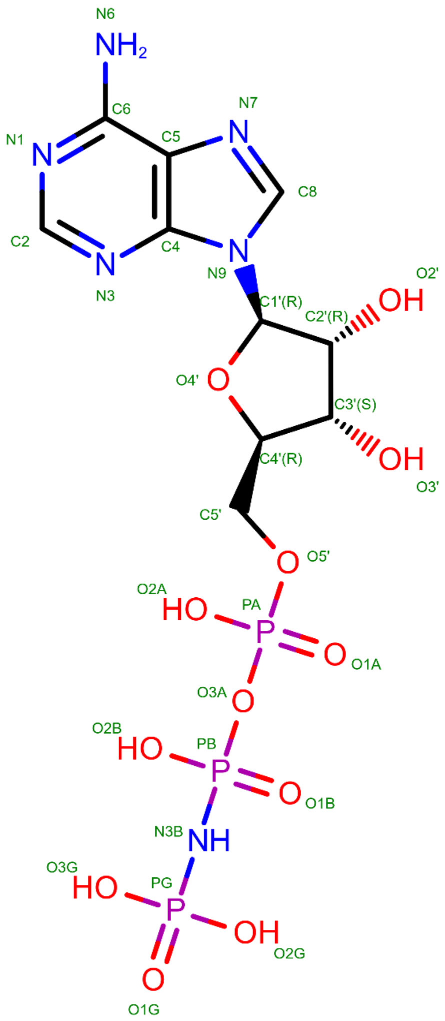
N6
NH2
C6
N7
N1
N
C5
N
C8
C2
C4
N
N
N3
N9
O2'
C1'(R)
C2'(R)
OH
O4'
O
C3'(S)
C4'(R)
OH
O3'
C5'
O
O5'
O2A
HO
PA
P
O
O1A
O3A
O
O2B
HO
PB
P
O
O1B
N3B
NH
O3G
HO
PG
P
OH
O2G
O
O1G

ANP

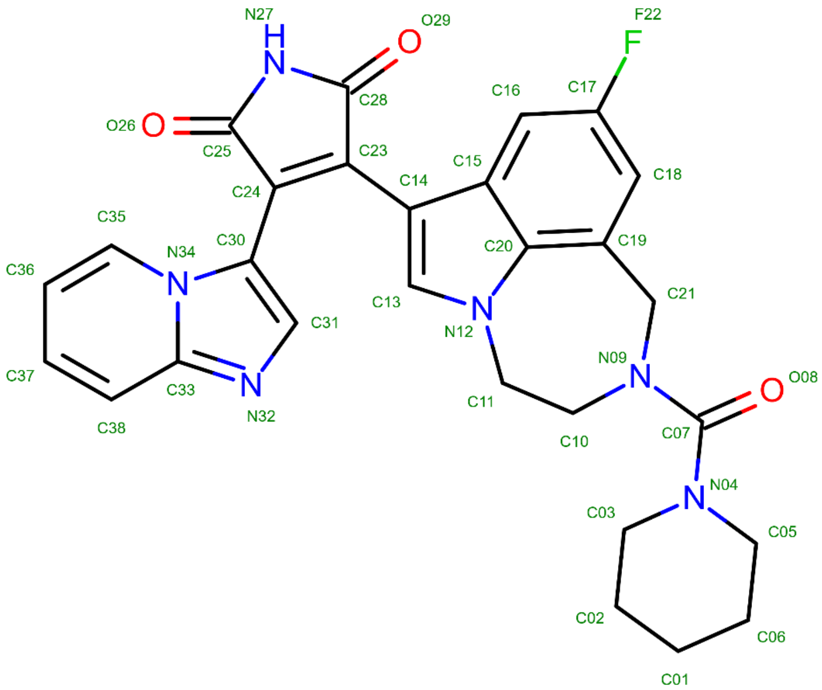
N27
O29
F22
C16
C17
O26
C28
C25
C23
C18
C15
C14
C24
C35
C30
C20
C19
N34
C36
C21
C13
N12
C31
N09
O08
C37
C33
C11
N32
C07
C38
C10
N04
C03
C05
C02
C06
C01

A1IYF

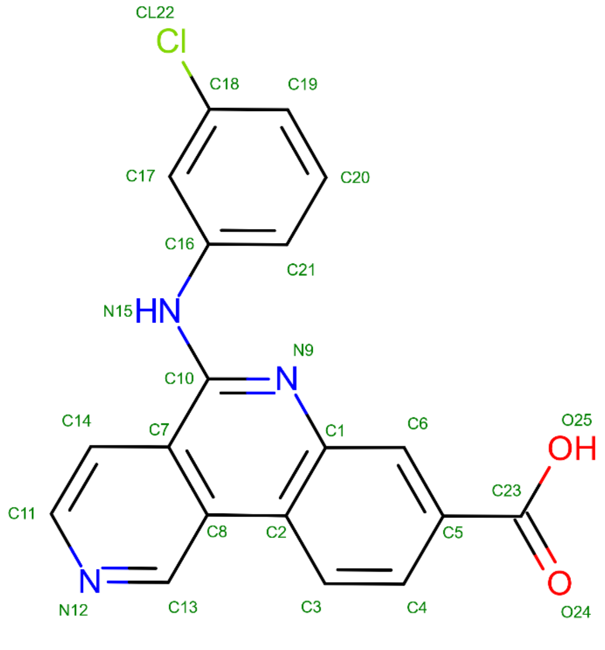
CL22
Cl
C18
C19
C17
C20
C16
C21
N15 HN
N9
C10
C14
C7
C1
C6
O25
OH
C23
C11
C8
C2
C5
N
O
N12
C13
C3
C4
O24

3NG

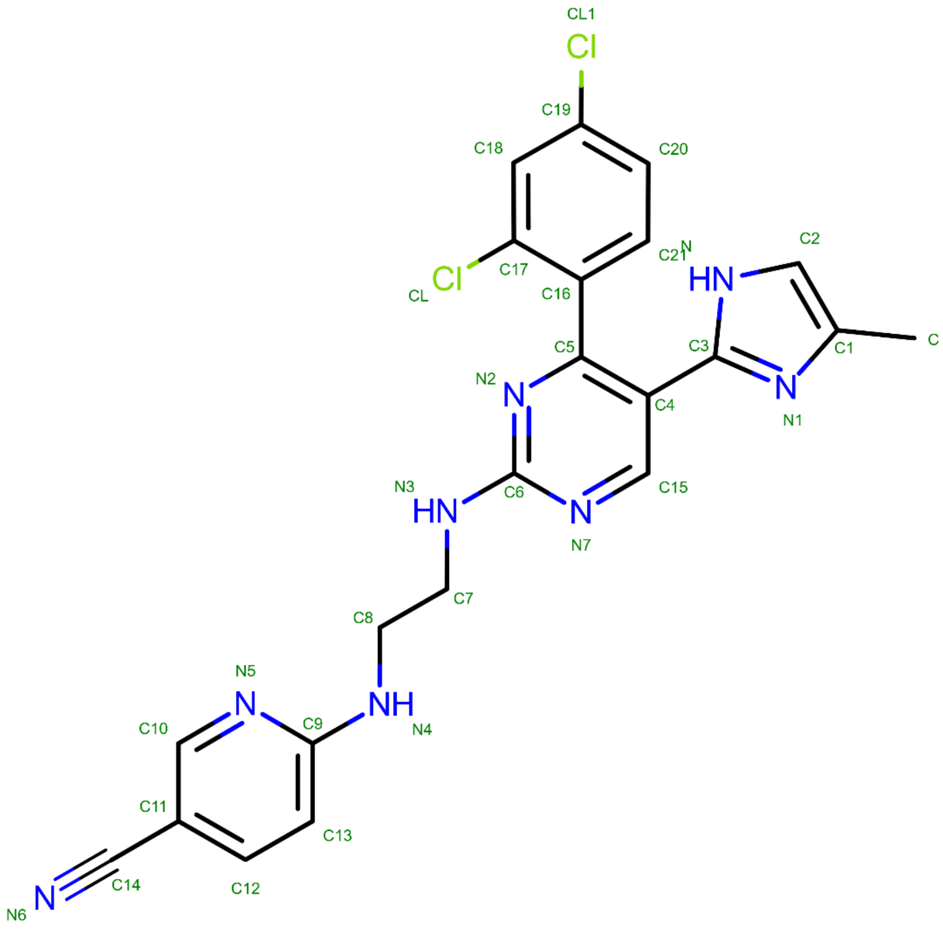

65C

S2. Top five co-crystallized GSK3β ligands (ADP, ANP, A1IYF, 3NG, and 65C).

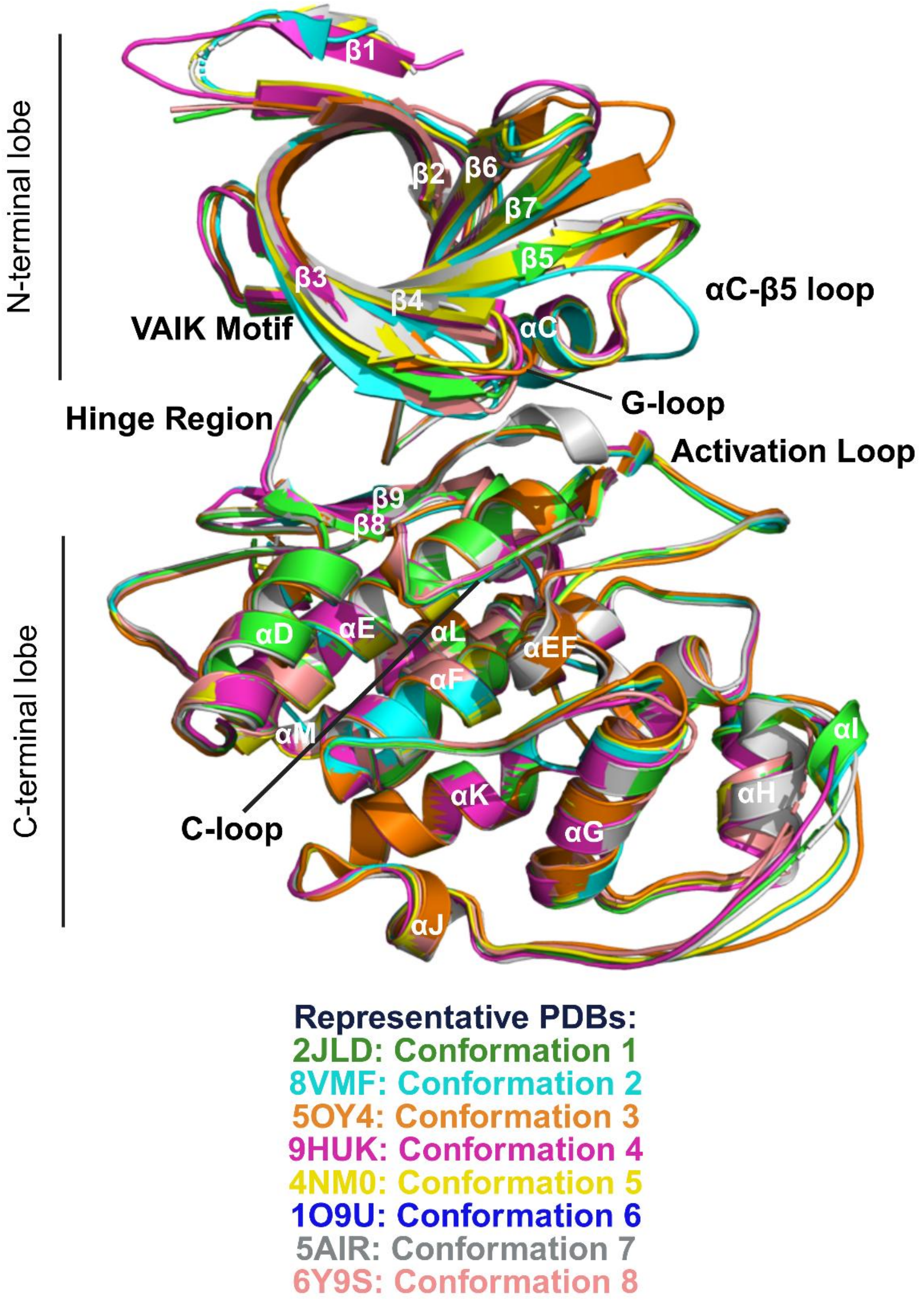

S3. Eight representative conformations of GSK3β with overlaid PDB IDs.

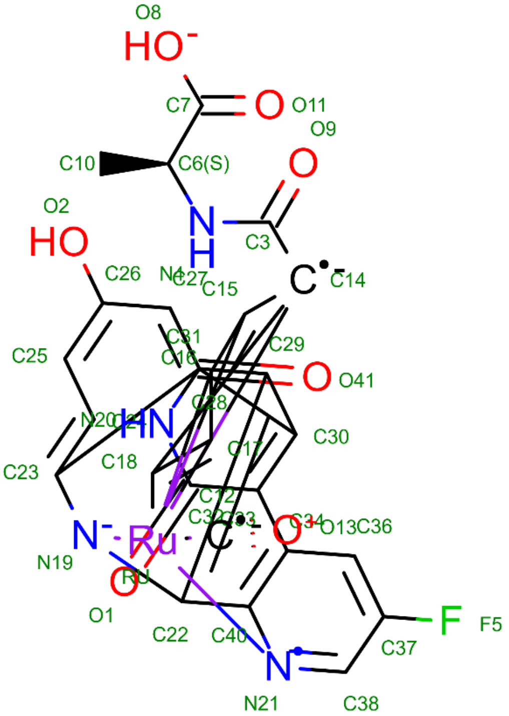

O8
HO⁻
C7
O
O11
O9
C10
C6(S)
O
N
H
C3
O2
HO
C26
N4
C27
C15
C14
C25
C31
C16
C29
O
O41
C28
N20
HN
C30
C23
C18
C17
C12
C32
C33
C34
O13
C36
N⁻
Ru
N19
O
Ru
O1
C22
C40
F
F5
C37
N
N21
C38

AG1

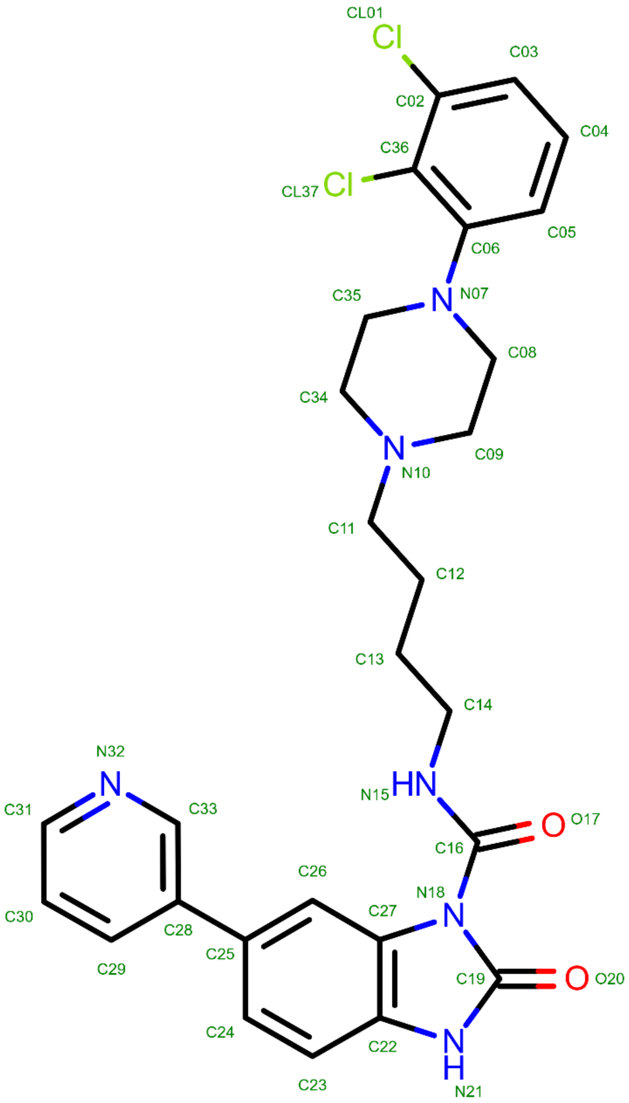
CL01
Cl
C03
C02
C04
C36
CL37
Cl
C05
C06
C35
N
N07
C08
C34
N
C09
N10
C11
C12
C13
C14
N32
N
HN
N15
O
O17
C31
C33
C16
C26
N18
C27
N
C30
C28
C25
C19
O
O20
C29
C24
N
H
C22
C23
N21

A1IXL

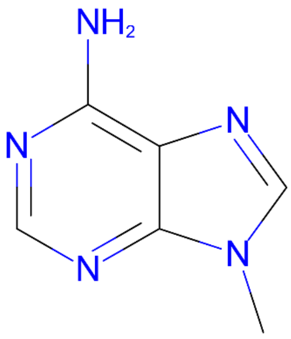

ADZ

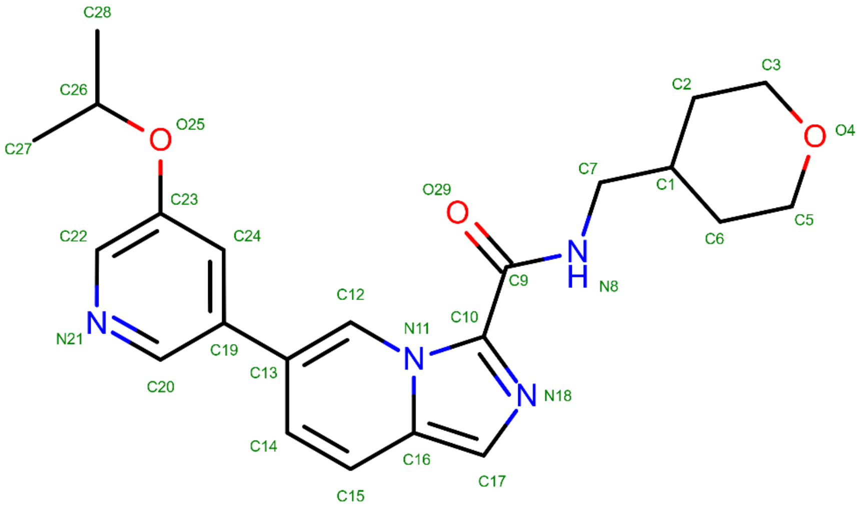

OHK

S4. Representative Type I inhibitors for GSK3β Conformations 1, 4, 6 and 8 (AG1, A1IXL, ADZ, and OHK).

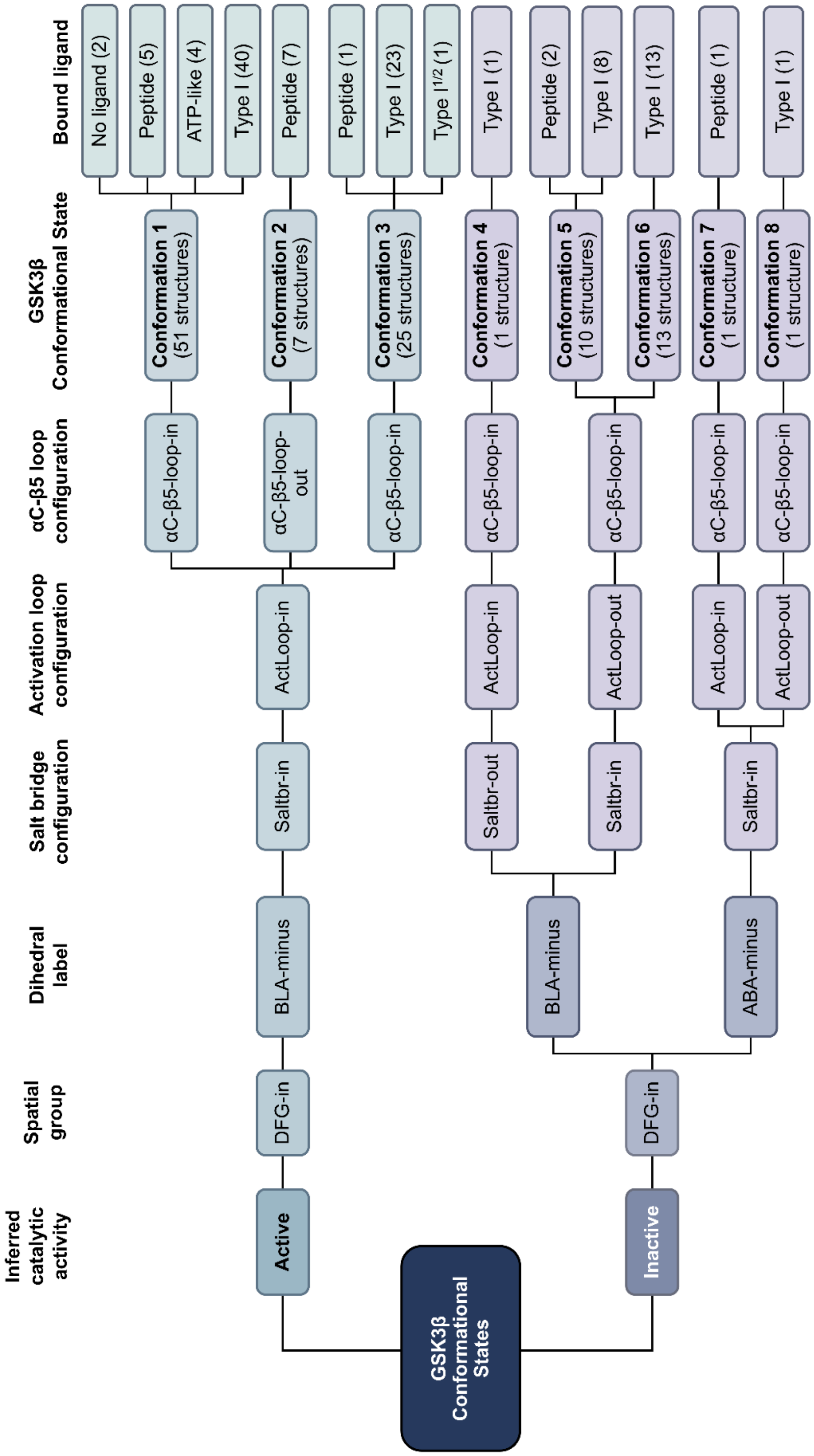

S5. Overview of GSK3β Conformational States.

Supplementary Table S1. Unique GSK3β PDB IDs (111).

4NM5A, 4NM7A, 4NM3A, 1J1BA, 1J1CA, 8XN6A, 1PYXA, 9X2QA, 9X2UA, 9X2WA, 9X2XA, 9X2YA, 1GNGA, 1H8FA, 1I09A, 7U2ZA, 6HK4A, 5F94A, 5F95A, 7U31A, 7SXJA, 7OY5A, 7U33A, 7U36A, 2OW3A, 3GB2A, 6TCUA, 5T31A, 7Z1GA, 7B6FA, 8AUZA, 4AFJA, 3ZRKA, 3ZRLA, 3ZRMA, 5K5NA, 1R0EA, 1Q5KA, 3I4BA, 3L1SA, 2JLDA, 3PUPA, 6B8JA, 6H0UA, 6V6LA, 3DU8A, 9FR5A, 9FR6A, 9FR7A, 9FR8A, 9FR9A, 9HULA, 9HV3A, 1Q3DA, 1Q3WA, 1Q41A, 1Q4LA, 3Q3BA, 4J1RA, 5HLNA, 5HLPA, 5KPKA, 5KPLA, 5KPMA, 4IQ6A, 3SAYA, 4PTCA, 4PTEA, 4PTGA, 8DJCA, 8DJDA, 8DJEA, 8FF8A, 3F88A, 4ACCA, 4ACDA, 4ACGA, 4ACHA, 5OY4A, 6Y9SA, 4NM0A, 9X2VA, 6HK7A, 7SXHA, 3F7ZA, 3SD0A, 3ZDIA, 4B7TA, 6HK3A, 6Y9RA, 1O9UA, 9PE9A, 7Z1FA, 8AV1A, 6GN1A, 2O5KA, 1UV5A, 6GJOA, 3M1SA, 9HUKA, 4J71A, 8QJIA, 4DITA, 8VMFA, 8VMGA, 8VMEA, 4NU1A, 5AIRA, 6AE3A, 9HVXA, 9HW6A

Supplementary Table S2. αC-β5 Loop Displacement Comparisons.

Stabilized GSK3β Conformation	PDB ID	Loop RMSD (Cα, Å)*	Loop Centroid Displacement (Å)#,$
2	*8VMF* (reference)	0	0
2	*4NM3*	1.877	0.751
2	*4NM5*	1.985	1.027
2	*4NM7*	1.932	0.870
2	*8VMG*	2.258	1.368
2	*8VME*	1.891	0.907
2	*4NU1*	1.901	1.707
1	*2JLD*	7.001	6.719
3	*5OY4*	7.765	7.400

*Local Root Mean Square Deviation (RMSD) calculated across backbone Alpha Carbons (Cα) of residues 89-94 after global structural alignment using the CE algorithm in PyMOL.

Linear distance measurement between the center-of-mass pseudoatoms of the target loop versus reference loop 8VMF.

$ The obtained centroid displacement distances were sorted into three classes: small (≤2 Å), medium (between 2 Å and 4 Å), and large (≥4 Å) as previously describe (van Westen et al., 2010).

Supplementary Table S3. GSK3β Observed Conformations Frequency.

Stabilized GSK3β Conformation	Count	Observed Frequency
1	51	46.8 %
2	7	6.4 %
3	25	22.9 %
4	1	0.9 %
5	10	9.2 %
6	13	11.9 %
7	1	0.9 %
8	1	0.9 %

Supplementary Table S4. Representative Type I Ligands Summary

Stabilized GSK3β Conformation	Ligand ID	Core Chemical Scaffold	Key Metric Altered
Conformation 1	AG1	organoruthenium half-sandwich complex	BLA-minus, salt-bridge and activation loop intact.
Conformation 4	A1IXL	2-oxobenzimidazoline	BLA-minus, salt-bridge broken.
Conformation 6	ADZ	Minimal Purine	BLA-minus, activation loop displacement and Tyr^{216} phosphorylation.
Conformation 8	OHK	imidazo[1,5-a]pyridine-3-carboxamide (IMID 2)	ABA-minus, activation loop displacement and Tyr^{216} phosphorylation.

Accompanying supplementary excel document includes 1) Raw KinCore Data, 2) Data Inclusion and Exclusions 3) Categorized GSK3β PDB Conformation & States.

References

van Westen, G. J., Wegner, J. K., Bender, A., Ijzerman, A. P., & van Vlijmen, H. W. (2010). Mining protein dynamics from sets of crystal structures using "consensus structures". *Protein Sci*, *19*(4), 742-752. https://doi.org/10.1002/pro.350
